# “I’m not an anxious person”: end-of-life care workers constructing positive psychological states

**DOI:** 10.1186/s40359-024-01885-5

**Published:** 2024-08-09

**Authors:** David Matthew Edmonds, Olga Zayts-Spence

**Affiliations:** School of English, The University of Hong Kong, Hong Kong, SAR China

**Keywords:** Mental health, Discourse analysis, Sociolinguistics, End-of-life care, Psychological states

## Abstract

**Background:**

Mental health is an issue of social and economic importance. Sociocultural and scholarly attention has largely focused on the negative aspects of mental health. That is, on mental disorders and illness and how they adversely impact our lives. In contrast, this paper forms part of a recent alternative empirical perspective in discourse-based research, by focusing on the positive aspects of mental health. In this article, we investigate how end-of-life care workers construct their positive psychological states.

**Methods:**

Our data are 38 audio-recorded and transcribed semi-structured interviews with end-of-life care workers from Hong Kong and the United Kingdom. We utilized thematic analysis to identify common categorial strands across the data and discourse analysis to identify the linguistic strategies that these interviewees used to talk about their mental health.

**Results:**

Our thematic analysis generated a superordinate theme across the interviews—namely, that of end-of-life care workers talking about their positive psychological states. We identify three generic ways that end-of-life care workers talked about these psychological states; by “foregrounding the positive,” “reformulating the negative,” and “dismissing the negative.” Our analysis also explicates how interviewees connected social and organizational support to being a benefit to their psychological states.

**Conclusions:**

Our work contributes to existing discourse-based and sociolinguistic research on mental health by turning their focus towards a consideration of its positive dimensions. We also identify recurrent linguistics strategies used by people to construct their mental health. Our analyses point to the importance of investigating mental health as a multidimensional concept that considers participants’ own reflections on their mental health.

**Supplementary Information:**

The online version contains supplementary material available at 10.1186/s40359-024-01885-5.

## Background

In recent years, organizations have raised awareness of mental health issues. Sindoni (2020) showed how *Beyond Blue* (an Australian charity) launched numerous online campaigns to promote positive messaging around mental health [1]. Such awareness campaigns encourage us to be open with our problems, talk to others, and seek help [2, 3]. For example, the Hong Kong government initiated the *Shall We Talk* campaign to encourage people to be open about their mental health problems by talking to others [4]. Some campaigns foreground the role of language—whether that be the labels used to describe our mental health problems, or, the very fact that we should *talk* about them to others [1, 5]. Ultimately, these campaigns are based upon the notions that talking to others has benefits and lowers stigma, and that in turn, leads to improved mental health [2].

Social science research has followed the lead of these awareness campaigns and has recognized the role of language in relation to mental health. Qualitative research in linguistics, sociology, and communication has focused on the discursive construction of mental health [6–9]. These studies have mainly focused on mental ill health. That is, on the *negative* aspects of mental health. By the “negative” aspects, we mean that such studies have investigated how people construct mental *illnesses* such as depression, anxiety, schizophrenia, and their *adverse* impacts on people’s lives [10]. These adverse impacts could include unemployment and relationship problems. In contrast, there is little research addressing the *positive* aspects of mental health, such as how people construct mental *wellness* and *positive* psychological states [11–14].

Relatedly, mental health is a spectrum encompassing both negative and positive aspects [10]. The Centers for Disease Control and Prevention (2021, para.1) defines mental health as encompassing “emotional, psychological, and social *well-being*. … it also helps determine how we handle stress, *relate* to others, and make *healthy* choices” (our emphasis). [15]  Thus, mental health is a spectrum with “thriving [and] surviving” on the one end, to “struggling or in crisis” at the other [16] (para.1). This paper focuses on the positive side of the spectrum, by investigating what we term *positive psychological states*. This term conceptualizes positive mental health as including positive emotions, positive dimensions of mental health, and resilience. Emotions and positive mental health fit under this term because of the well-established conceptual, theoretical, and empirical links between emotions and mental health [10, 13, 17–20]. In fact, some existing definitions of mental health include emotional states [20]. In addition, people themselves treat emotions and mental health as interrelated. During our interviews, we asked participants about their mental health and their answers often included descriptions of their emotions.

To examine these matters, this paper focuses on a population that one might not normally associate with happiness and positive emotions—those working in EOLC. That environment is not an easy environment in which to work, as studies have shown that these workers face psychological adversity in their work, including burnout, compassion fatigue, and other psycho-emotional problems [22–26]. These problems were compounded during the pandemic (when our interviews took place). [27] During this period, these people became frontline workers and dealt with death, restrictions, and poor mental health [28–30]. In line with the gap in discourse-based research, we examine how EOLC workers in Hong Kong and the UK construct their positive psychological states. We also aim to understand the insights that our findings can provide to an understanding of how people construct the positive aspects of their mental health more generally.

## Literature review

### End of life care during COVID-19

The COVID-19 pandemic hit the healthcare sector hard. Medical systems were pushed to the brink of collapse [31]. EOLC was no different, for example, with hospices tightening visitation regulations, employing video-mediated communication for families and healthcare professionals, and staffing shortages [32–34]. In the UK, hospices shut their doors to visitors and new patients, as well as having their care compromised due to resource and staff shortages [35]. EOLC services in Hong Kong also instituted limits on visits from family members for patients during the pandemic [28]. There was an added complication in Hong Kong, whereby everyone faced the possibility of forced government quarantine for up to 21 days if they tested positive. [36] Such upheaval was not dealt with easily dealt by EOLC workers, as they reported experiencing high rates of anxiety and depression during the pandemic [28]. However, studies have documented that not all EOLC workers reported negative experiences during the pandemic, with some valuing the increased time spent with patients and the collaboration with other sectors of the healthcare system. [25, 37] Our work fits within these existing studies of EOLC workers’ experiences during the pandemic. We extend this literature by applying a discourse analytic perspective to undertake a fine-grained examination of how these workers talk about their psychological wellbeing.

### Discourse-based studies of mental health

There is an established body of discourse-based research on mental health^1^. [38] These studies can be micro-analytic— by exposing the linguistic strategies used to talk and write about mental health. These studies can also be macro-analytic by considering what these constructions might tell us about mental health in society and media. Georgaca (2014) delimited four general strands of research on mental distress that use discourse analysis. [39] Namely, the constructions of mental health from those accessing services, healthcare professionals’ “professional accounts and practices” in clinical settings (p.56), media portrayals of mental health, and critical interrogations of clinical diagnoses and labels [39] (p.56–58). Our study fits within the second strand of studies by examining healthcare professionals’ reflections about their *own* mental health in relation to their work.

Discourse-based research shifts the focus away from mental health categories and labels as “explanatory resource[s]” and instead to “topic[s] to be explored” in their own right [40] (p.348). Thus, the focus is not on investigating conditions such as obsessive-compulsive disorder as an explanation or cause of behavior, but instead on explicating how people construct these conditions. These studies also center on the use of language (both written and spoken). Discourse-based studies explicate the “action orientation” of the language people use when talking or writing about mental health [41] (p.48). That is, the focus is on what such talk is *doing* in different contexts. Edwards (1999) analyzed how couples in counselling sessions described negative emotions to construct a troubled relationship and undermine their partner’s claims. [42] Such micro-analytic studies take a fine-grained examination of the functions of linguistic practices in particular contexts. In contrast, macro-analytic studies investigate how people’s constructions of mental health are tied to broader socio-cultural matters. Challenor et al. (2021) reviewed the work of Dlodlo (2018) as an example of how business professionals’ constructions of the mental health of other people was tied to the idea of employability. These constructions we also linked to the neo-liberal notions of “productive” and adaptable workers (p. 112) [43, 44]

These discourse-based studies have taken a negative focus in regards to mental health [14]. For example, Hung et al. (2023) documented how healthcare and social workers constructed the “psychological pain” that their jobs caused them [45]  (p.233). We do not know exactly why discourse-based studies have focused primarily on the negative dimensions of mental health. Yet, one possible reason could be the prevailing socio-cultural assumption that mental health is negatively defined [46, 47]. What remains less well understood is how mental health is talked about in positive terms.

### Turning to the positive side of mental health

In a review of the research of mental health portrayals in the media (including discourse-based studies), Atanasova and colleagues (2019) noted that “recent studies have focused on… ‘the illness side’ of mental health” [11] (p.1). Yet, the authors also noted that studies have begun to focus on “the wellbeing side” [11] (p.2). That is—while a gap exists—discourse-based studies are increasingly focusing on the positive aspects of mental health. Crucially, these studies focus on the discursive construction of positive psychological states as opposed to traditional qualitative examinations of the positive *outcomes* of psychiatric interventions.

Some studies have explored how people construct and define the very notion of positive mental health itself [48–50]. For instance, Chang et al. (2022) showed that psychotherapists defined positive mental health as consisting of “acceptance…normal functioning and thriving in life…resilience…absence of negative emotions…” [51] (p.1). Thus, one implication of this finding is that negative mental health is still defined by the absence of negative attributes. Our analysis also addresses how people construct positive psychological states by dismissing and reformulating negative descriptions.

Another strand of research has focused on how people construct positive mental health during crisis times [14, 52]. For instance, Bullo et al. (2022) examined qualitative responses to a survey of people living in the UK during the COVID-19 pandemic lockdowns [12]. They found that people reported ‘looking for the best’ in such challenging times and also reported that they derived happiness from a range of sources. Relatedly, Raza et al. (2023) interviewed people who were infected with COVID-19 at the beginning of the pandemic and found that despite the challenges they faced, the interviewees often redefined mental health struggles “in a positive and adaptive way” [53] (p.123). Our study also identifies the different ways that people reformulate their positive psychological states but extends its focus to a different population—people working in EOLC.

## Methodology and data

The analysis sits within a broader social constructionist framework, which focuses on how people talk about their experiences through various discursive means [8]. Social constructionist approaches have long influenced discourse-based research on mental health [8, 14]. Our analytic stance is that we acknowledge that people’s mental health experiences are real for them, but we remain interested in how they discursively construct these experiences in the interviews [54]. In following a social constructionist approach, we acknowledge that we cannot examine their actual psychological states either at the time of the interview or during the pandemic. Instead, we examine their reflections and accounts of the psychological states that they report experiencing during their work and the pandemic.

The data are 38 interviews with EOLC workers who worked in the specialty in Hong Kong (16 interviews) and the UK (22 interviews). The interviews ranged from 14 to 39 min in length. The gender ratio of interviewees was reasonably balanced in the Hong Kong sample (7 males and 9 females). However, the UK sample was skewed heavily towards females (3 males, 19 females). In Hong Kong, our interviewees worked in clinical, administrative, and academic positions. The UK interviewees worked in clinical, administrative, research, and pastoral positions. In both samples, we interviewed workers from hospice, hospital, community, and non-governmental organization (NGO) settings. We were liberal in our inclusion criteria for participants. Namely, they had to simply work in a position in a setting that was involved in EOLC. This broad focus ensured that we gained insights into as diverse a range of experiences as possible from those working within EOLC.

The purpose of this study is not to compare cultural differences in EOLC workers’ experiences. Nevertheless, it is worth mentioning some of the connections (or not) between the contexts of our samples. These connections also provide justifications for examining interviews from the two contexts together. The UK and Hong Kong share similar medical systems. [55] Indeed, the Hong Kong health system was directly shaped by the National Health Service in the UK. [56, 57] Previous research has noted that the health systems of Hong Kong and the UK are broadly comparable [56, 57]. Furthermore, EOLC in Hong Kong—originally in the form of hospice care—was founded and developed by the British. [58, 59] Despite these similarities, differences between the two jurisdictions exist. Hong Kong has much less developed EOLC policies and services compared to the UK [55, 58, 60]. In addition, comparison between the two contexts is complicated because the jurisdictions have different definitions as to what constitutes ‘end-of-life.’ [61] In Hong Kong, it is defined as months at most [62]. Whilst, in the UK, it is defined as those patients who will die in the course of 12 months [61–64]. We acknowledge the differences that exist between the two EOLC systems. Yet, we treat cultural differences and how these might be articulated in EOLC workers’ narratives as beyond the scope of this paper.

The interview questions were derived by the research team for this study. The interview questions (in English) used in this study can be found in the online supplementary material for this article. The focus of the interview schedules was slightly different. The UK interviews focused on working during COVID-19 and the Hong Kong interviews focused on working in EOLC more generally. Nevertheless, the Hong Kong interviews were conducted during the pandemic and inevitably the participants’ answers touched on their experiences during this tumultuous period. As a result, much of the content of both sets of interviews was similar, and thus, we treat them as broadly comparable.

We recruited participants through purposive sampling—recruiting anybody who worked in EOLC in the UK and Hong Kong healthcare, social work, and NGO sectors. For participant recruitment, we promoted our study online through social media and posts on selected websites and newsletters. We directly contacted organizations to get them to promote our study to their staff. In addition, we utilized snowball sampling whereby interviewees recommended further prospective participants. Ethical approval for the study was granted by the authors’ university [EA210454]. All interviewees provided written informed consent. Interviews were conducted online over Zoom or in person and were audio recorded. The interviews with Hong Kong healthcare professionals were conducted in English or Cantonese (the variant of Chinese spoken in Hong Kong)^2^. The interviews with those participants from the UK were only conducted in English. The interviews were then transcribed in full. Interviews in Cantonese were translated into English by multilingual members of the research team. Six interviews were conducted in Cantonese. However, only English interviews are the focus of this paper. The reason for this focus is that we wanted to ensure that we were consistent in comparing equivalent ways of talking about mental health. In addition, both authors do not speak Cantonese and thus lack the requisite proficiency to engage with the data at a micro-analytic level. Finally, the focus of this paper is not on examining cultural differences in how mental health is talked about.

Thematic analysis (TA) was utilized [65] alongside discourse analysis (DA) [40, 41] to analyze the data. First, TA was used to map and organize the dataset, assisted by NVivo. [66] TA provided a way to find the common thematic threads across the interviews [65]. Indeed, TA involves “identifying, analysing and reporting patterns (themes) within data” [65] (p.75). TA began with the first author reading all the interview transcripts numerous times. During this process, notes and analytic memos were made. [65] The interview transcripts were coded line by line by the first author. Coding was largely deductive and was guided by an existing interest in mental health, emotions, and psychological wellbeing. Codes were also generated from an inductive reading of the data. The coding did not use a pre-determined codebook, nor was inter-rater reliability calculated. [67] These decisions were made because TA acknowledges that analysis is a “subjective” and an “interpretative reflexive process” [67] (p.333–334). The transcripts were coded in an attempt to generate common broader level patterns of meaning—that is, themes—across the corpus [65] (p.82).

The coding focused on parts of the transcripts where interviewees used mental health descriptions (e.g., “anxious” and “burnout”) and on their answers in response to explicit questions about their mental health. During coding, it was noticed that participants also included emotion descriptions (e.g., “happy,” or “stressed”) in their responses to questions about their mental health. As such, these portions of the transcripts were also coded in depth. The codes were then sorted into overarching thematic categories.

The analysis in this paper is structured around one superordinate theme—that of *positive psychological states*.

Following the initial use of TA, DA was used as a subsequent analytic tool. Specifically, DA was used to further develop the superordinate theme—namely, by identifying the specific linguistic practices that interviewees used to talk about their psychological states [68, 69]. DA was also used to identify the “action orientation” of interviewee’s talk [41] (p.48). The first author conducted a DA on all the interview excerpts contained under the superordinate theme. The extracts that are presented below have been anonymized and any identifying information has been replaced with pseudonyms. The lines in the excerpts that are of analytic interest have been bolded. The selected extracts are representative of the other cases included in the superordinate theme and of the other instances across the dataset. The clearest cases to illustrate the analytic argument have been presented.

## Analysis

This paper considers how EOLC workers talk about their positive psychological states. The analysis begins by explicating three generic ways that the interviewees talked about their positive psychological states—namely, by “foregrounding the positive,” “reformulating the negative,” and “dismissing the negative.” Finally, we explore how the interviewees constructed “social support” and “organizational support” as crucial components of improving their psychological wellbeing.

### Foregrounding the positive

One way that EOLC workers talked about their positive psychological states was by describing them. The first extract shows a participant (PAR) detailing the personal lessons that she gained from working in EOLC during the COVID-19 pandemic. The participant foregrounds her “resilience” while working amidst such tense times.

### Excerpt 1: UK interview 7

**Table:** 

01	PAR:	I’d like to think it’s taught me **how resilient I am**, but in fairness, **I knew**
02		**how resilient I was before** because I’ve had a very- my life hasn’t been
03		without it’s complications. Do you know what I mean?
04		*((a few lines of identifying information removed))*
05	PAR:	So, **I know that I’m resilient.** I think because of that I have developed a lot
06		of coping strategies that then play into things at work
07		I think it reinforced some of those things that I knew were there already

The participant’s answer is in response to a question from the interviewer about how the pandemic affected her mental health in positive ways. Despite the question potentially making relevant an answer about positive psychological states, the participant could always have talked about her negative psychological states. Nevertheless, the participant’s answer includes the use of what one might call a *positive mental health descriptor*. That is, the interviewee describes her “resilience” gained from working during the pandemic and that consolidated her existing strengths. Resilience is a psychological concept that is defined as the “positive adaptation, or the ability to maintain or regain mental health, despite experiencing adversity” [70] (p.259). This participant’s unpacking of what she means by “resilience” falls into such a “positive adaptation[s]” definition, as she describes it as a rich set of “coping strategies”. Based on the sequential context in which it occurs (following the question about positive psychological states), we can understand that the interviewee is using “resilience” to describe her positive state. Indeed, her use of this descriptor “resilience” implicitly undercuts any presumption of the pandemic negatively affecting her by foregrounding her positive mental health.

The interviewee repeats the word “resilience” three times in this extract. This repetition serves to emphasize the interviewee’s point about this quality of resilience. On the other hand, it is reflective of how the interviewee made sense of her *present* psychological states in relation to what she had gone through in the past. Namely, this interviewee alludes to previous difficulties in her life (lines 02–03) and that this is bound up with her “resilience” being a persistent personal trait that continues to aid her (“because of that I have developed a lot of coping strategies”). Thus, in temporal terms, her positive psychological states are related to an ongoing characteristic of hers.

The repetitions also occur when she is describing a personal quality (“I am resilient”). In this extract, the interviewee accomplishes self-praise with her talk, by illustrating her own personal strengths. In face-to-face interaction, self-praise is a delicate matter that is downplayed and mitigated. [71] In contrast, in this case, the praise of her own coping abilities is direct. Alongside this repetition, she indexes that she herself is the agent of “develop[ing]” these strategies. In all, these linguistic practices accomplish the overarching interactional activity of constructing a positive version of herself in the interview.

The second excerpt is from an interview with a Hong Kong EOLC worker, wherein he describes the “good feeling” and “energy” that he gets from his job.

### Excerpt 2: Hong Kong interview 1

**Table:** 

01	PAR:	They are willing to talk to you and trust you. I think this is quite, **it makes me**
**02**		**have quite a good feeling because you have been trusted.** So, I think we
03		can build quite a good relationships with the patient and the family when they
**04**		**trust you. So**,** I think this is some kind of energy. Gives me energy to**
**05**		**continue.**

In this excerpt, the interviewee uses the term, “good feeling,” to describe a positive psychological state. The term is generic in reference—namely, it positively assesses a “feeling” as “good” but does not ascribe a specific psychological state (for instance, “happiness”). In this case, we treat “feeling” as equivalent with “emotion”. Indeed, “in everyday parlance, the word feeling is frequently used as though it is synonymous with emotion” [72, 73] (p.522). This state is linked to a specific cause—namely, a “good relationship” and the interpersonal “trust” that the interviewee fostered with his patients and their family members. In this instance, the interviewee constructs a ‘transference effect’, whereby the positive interactions with patients and families have bolstered his psychological wellbeing. Thus, the interviewee highlights the mutuality of these relationships as a rewarding part of EOLC. This extract provides a contrast to the previous case, whereby in the latter the positive psychological state was tied to his own actions. In this case, the positive psychological state is derived—at least in part—from interactions with others.

The interviewee follows by initially characterizing “this”— referring to “good relationships”—as “some kind of energy.” Yet, he then reformulates to a causal attribution, that these relationships induce a psychological state of “energy.” Importantly, such “energy” allows him to continue his work in the face of (implied) adversity. The interviewee uses the “emotions as energy” metaphor to describe his psychological states, which has been noted as a common way for people to communicate their emotions to others [74] (p.333). The EOLC worker treats “energy” as a positive psychological force that propelled him forward in his workplace.

### Reformulating the negative

In our interviews, EOLC workers did describe the negative psychological states that they had, which was manifest in descriptors such as “anxious” and “worried.” Nevertheless, our interviewees also reformulated these negative psychological states in positive terms. The excerpt below provides an instance of this, as the interviewee begins by labelling some of these typical negative psychological states, such as “worried,” “tearful,” “low in mood,” and “anxious.” However, the interviewer issues a follow-up question (line 09) that shifts the discussion to both a new temporal frame (“coming out of the pandemic”) and new psychological states (“your mental health…is starting to get better”). This question provides the interviewee with the opportunity to recast her psychological state in a positive light.

### Excerpt 3: UK interview 9

**Table:** 

01	PAR:	I found the more that we went into the pandemic, the more I was starting to
02		**feel anxious**. **I was anxious** about other people’s behaviors and how that
03		inflicted upon me and my family. **I worried** about how people in the general
04		public felt that I was as a ((*job title*)) and my people and so many people were
05		dying. What did people think about our skills and things? And I sort of felt
06		embarrassed because I had to say no.
07		((*one line of identifying information removed))*
07		**But I felt anxious. I felt tearful. I felt withdrawn** and just **low in mood**
08		*((11 lines of talk about support from colleagues removed))*
09	INT:	Have you found that now that you’re sort of coming out of the pandemic,
10		your mental health in relation to your work is starting to get better?
11	PAR:	Absolutely. **So when I was on the antidepressants**,** it gave me time to**
12		**reevaluate things.** And as we started to come out through the other side and
13		things started to open again, I decided that at my age that I’m heading nearer
14		towards retirement than I’m not. And I had the opportunity to change, to not
15		just do a job that I love anyway, but **to really do something that I enjoy**
16		within that job, if that makes sense.
17		And it just gave me a chance to reevaluate my life. And I decided to go part
18		time, a different role within the organization.
19		And as things started to improve and open up as a society,
20		I was able to get out and about more, and **I just felt much better for it**.

The first part of the excerpt comes from near the beginning of the EOLC worker’s answer to a question about her emotions during the pandemic. The interviewee attributes her negative psychological states to various stressors, including the initial fear of COVID-19 infection (lines 02–03) and the public’s attitudes towards healthcare professionals at the time (lines 03–04). Following the interviewer’s question, the EOLC worker’s narrative shifts. Namely, the use of “antidepressants” (line 11) marked a change in this EOLC worker’s psychological states. In particular, the medication allowed the interviewee to “reevaluate things” (line 12). Based on the positioning within the narrative’s wider structure, the antidepressants are constructed as a solution to the interviewee’s just-described negative psychological states. The antidepressants are a turning point, offering new temporal affordances—namely, providing the interviewee with a period of cognitive deliberation (“reevaluate my life”) and psychological stability.

The interviewee draws on a recovery frame in her narrative [75, 76]. That is, the interviewee structures her narrative around an initial affliction of “anxiety” and poor “mood,” which was then overcome to result in better mental health. The interviewee reformulates her psychological states in a positive manner, by stating how she “felt much better for it” (line 20). While the interviewee constructs her positive psychological states as (somewhat) occasioned by her use of antidepressants, she also implicates her own agency and other contextual factors as playing a part. The mental stability offered by the medication allowed her to make concrete changes in relation to her employment (lines 12–18). She also attributes contextual factors such as her increased “age” (lines 13–14) as contributing to this change. Thus, the antidepressants are not positioned as the sole “cure” [77] for the interviewee’s psychological states. Rather, the interviewee attributes the medication as one of several causes, alongside her new role, and the temporal development of the pandemic itself [12]. As such, this excerpt also points to the fact that this EOLC worker does not construct her psychological states in a vacuum. Of course, she constructs and positions her experiences in relation to the socio-cultural particulars of the time—in this instance, the COVID-19 pandemic.

The excerpt below is from an interview with an EOLC worker in Hong Kong. The interviewer issues his “last question,” which is formulated as summarizing one upshot of the EOLC worker’s comments throughout the interview (lines 01–03). Namely, that the EOLC worker has had more “positive[s] than negative[s]” states while working in the field. The interviewer’s follow-up question (line 05) prompts the EOLC worker to elaborate on the reasons for the positive states outweighing the negative (lines 06–14).

### Extract 4: Hong Kong interview 3

**Table:** 

01	INT:	And my last question is, altogether, what we’ve discussed, it sounds
02		like you’re saying that working in palliative care, there are much more
03		positives than negatives. Would you say that that’s true?
04	PAR:	Yes, I think so.
05	INT:	Okay. And why do you think there’s more positives than negatives?
06	PAR:	**Hmmmm**,** because I think why it is more positive than negative** is there
07		is- that is palliative care is working with the patient and relative, patients
08		who are terminally ill and facing death and dying. I think it’s more positive.
09		**Everyone will die. Everyone will face this problem in one day.**
10		Then I have the chance to learn more, to understand more about death and
11		dying and contact with others who are not working in this. So, I will think
12		that when this day comes, **I will be less anxious.**
13		Or, when this day comes to my family member,
14		**I will be less worried**,** less afraid.** Something like this.

The interviewee begins by describing what her job involves, in simple terms, “working with…patients who are terminally ill and facing death…” This description carries negative connotations—namely, one might expect working with dying people to be a difficult job that not everyone would be able to handle. Nevertheless, this is immediately contrasted with the explicit “positive” assessment (line 08). The interviewee’s next statement refers to the inevitability of death, “everyone will die,” and negatively assesses such a fate by evaluating it as “this problem.” Thus, at this point in her answer, the EOLC worker has explicitly and implicitly constructed aspects of her job in a negative manner.

The remainder of the EOLC worker’s answer constructs her psychological states as positive—or at the very least, less negative. She describes the opportunities afforded to her by her job around “the chance to learn more, to understand more about death.” These opportunities have resulted in positive psychological states for her in relation to the prospect of her own death, or of those close to her. These psychological states are feeling less “anxious,” “worried,” and “afraid.” This excerpt forms a contrasting case to the previous one because this EOLC worker is not describing completely positive psychological states. Instead, the EOLC worker is describing (potential) psychological states as being *less negative*. We can still understand this case as an instance of “reformulating the negative” for four reasons. First, these descriptions are downgraded versions of adverse psychological states. For instance, if we think of “anxiety” as forming the ‘negative’ pole of a hypothetical scale of emotions, the quantifier “less” shifts the referent towards the more ‘positive’ end of the scale [78]. Second, these descriptions are included within the sequential context of an answer that explicitly casts the EOLC worker’s psychological states as *positive.* Thus, and third, the EOLC worker casts her (potential) psychological states in opposition to both explicit and implied negative ones. Fourth, the psychological states are also “reformulated” temporally as well. That is, the EOLC is describing her potential psychological states in relation to death in the *future* (“when this day comes”). The implication being that at present—or in the past—she may have negative emotions in relation to the prospect of death.

This case is similar to Extract 2 in terms of the relationality of EOLC. Specifically, these interviewees both link their positive (or less negative) psychological states as arising from the result of the relationships that they forged with patients and family members as part of their work. In particular, in this extract, the interviewee cites the benefits to her wellbeing (“less worried”) from “learn[ing]” through her work with terminally ill patients.

### Dismissing the negative

Another thematic strand in the interviews centered on how the participants constructed their positive psychological states in contrast to negative ones. Specifically, EOLC workers could outright dismiss the fact, or even the implication, that they had experienced any negative psychological states in their job. Two cases are shown below.

### Excerpt 5: UK interview 6

**Table:** 

01	PAR:	And for me personally I think- **I’m not an anxious person**, not in regards to
02		things like that and **I didn’t continue to be anxious** but I did notice a lot of
03		the team being anxious

### Excerpt 6: Hong Kong interview 4

**Table:** 

01	INT:	So, how does working in palliative care create problems for your mental
02		**health?**
03	PAR:	Oh, for myself?
04	INT:	Yeah for yourself.
05	PAR:	**Hmmmm.** ***((Long pause))*** **No.**
06	INT:	Okay.
07	PAR:	**Actually no- no problem.** I’m thinking a lot. *((laughs))*

Both interviewees use the discursive strategy of negative definition to describe their psychological states—namely, by employing “not *X*…” formulations. In excerpt 5, the interviewee defines herself as “*not* [an] anxious [person].” Specifically, this claim is tied to “things like that,” which refers to an earlier comment from the interviewee’s turn about the death of one of her colleagues and the negative impact that it had on her other teammates (not in the excerpt). This interviewee also uses this negative description to position herself in opposition to her colleagues, who remain “anxious” (line 03).

In the sixth excerpt, the EOLC worker is asked whether her job “creates problems for [her] mental health.” After a clarification (lines 03–04) and a long pause, she answers in the negative, “no”. Her subsequent turn is prefaced with “actually,” which has been shown to index that the content of a turn of talk pushes back on, or challenges, a claim made in another speaker’s turn [79]. The EOLC worker’s turn explicitly rejects the assumption in the initial question that her job “create[s] problems” for her psychological wellbeing. The EOLC worker also claims that she was trying to identify any examples of “problems.” This case contrasts that in excerpt 5 because it relates to the interviewee’s psychological state in relation to her job generally, and not specifically in relation to the pandemic. The pragmatic function of the ‘dismissing of the negative’ strategy is seemingly to imply positive (or at the very least, neutral) psychological states. In other words, the “*not* X…” constructions in these utterances imply the opposite of what is stated. In all, interviewees could define their psychological states in terms of what they were *not*.

### Support from others

Our thematic focus throughout the analysis has been largely individualistic. That is, on interviewees constructing their *own* psychological states. Yet, interviewees also constructed their psychological states in relation to more *interpersonal* matters. Specifically, the interviewees attributed the social support provided by their family, friends, and colleagues as beneficial for their mental health. For brevity, this section only focuses on the construction of support from *colleagues* in relation to their positive psychological states. The next excerpt illustrates an interviewee citing mutual support amongst colleagues as beneficial for his mental health.

### Excerpt 7: UK interview 5

**Table:** 

01	PAR:	Obviously, it’s that thing about team talk, about** how I support other nurses.**
02		We’ve got some clinical supervision and I think that’s really important I
03		think that’s really important **so that people can get it out there**,** talk about**
04		**the stress**, talk about that staffing issues, and **how they felt**. Sometimes
05		people have **bottled things up** and I think as we go out of it now, sometimes
06		the wheels might fall off for some people
07	INT:	**So workplace support was very crucial for everyone’smental health**?
08	PAR:	Yeah I think so from the simple one from any nurse at the end of the shift
09		saying **are you okay**, you alright, you being okay? You know and knowing
10		the people you work with. I think that’s the difficulty in some units, where
11		it’s all bank and agency, no one knows each other. And I think that’s a worry
12		because people then go off with awful things and no one checks that they’re
13		okay.

In the preceding question, the interviewer asked the EOLC worker, “so if we think about your mental wellbeing during the pandemic, how did working in end-of-life care create problems or stressors for you?” (not in the transcript). The interviewee’s talk in the excerpt above forms the later part of his extended answer. The interviewee’s answer details different forms of support, such as “team talk,” “clinical supervision,” and talking to one another about “stress” and “feelings” (line 04). This form of ‘talk as support’ can be understood as disclosure about one’s mental health state. The interviewee positively assesses the benefits of disclosure, “really important […] people can get it out there, talk about the stress.” The interviewee then contrasts disclosure with a lack of it by utilizing a “body as container” metaphor [74] (p.333)—namely, “bottling things up” (line 05). A lack of disclosure is linked to adverse consequences, “wheels might fall off,” with this idiomatic phrase referring to people psychologically ‘losing it.’ Through this contrast, disclosure is constructed as something beneficial in relation to psychological states. Disclosure is constructed as a way of overcoming suppression of one’s negative psychological states. The interviewer’s turn at line 05 provides further evidence for this claim. The interviewer’s turn presents the gist of the EOLC worker’s previous talk, which is that this mutual support had a positive effect (“crucial”) on “everyone’s mental health.” Finally, the interviewee’s construction of disclosure arguably reproduces socio-cultural norms around talking to others about mental health problems as a means of alleviating them [80, 81]. In particular, Elraz (2018) noted that disclosure is an act that can counter mental health stigma in the workplace [80].

After the follow-up question, the interviewee gives examples of what *opportunities* for disclosure might look like ‘on the shop floor.’ Namely, with colleagues asking one another if they are “okay” or “alright,” which would make it ‘conversationally relevant’ for colleagues to disclose any problems. The remainder of the interviewee’s talk in this extract (lines 11–13) implies that without support, his colleagues would continue to experience negative psychological states.

There is an intersubjective element that is constructed as central to this support. EOLC workers share the same circumstances and similar experiences by virtue of working together. In excerpt 7, the interviewee emphasizes the importance of “knowing the people you work with,” which was the case in his workplace. He draws a contrast to other workplaces, which may have outside healthcare staff sourced from other places (“bank” and “agency”). The interviewer evaluates it as less than ideal to have such employees in the workplace in terms of support because they do not have the same shared experiences (“no one knows each other, and I think that’s a worry”). Ultimately, it is through these intersubjective experiences that colleagues can understand and support one another in order to bolster one’s psychological state.

### Organizational support

Support not only comes from colleagues but also from workplaces. The organizations for which interviewees worked implemented a range of measures to provide staff with support for their mental health. Some organizations set aside designated rooms in the workplace where employees could go and relax. Interviewees also reported that during the pandemic, their workplaces set up regular Zoom video calls where employees could ‘catch up’ with one another. In Hong Kong, a formalized program existed whereby colleagues would join one another in regular face-to-face meetings and talk about their work experiences and mental health issues. Interviewees cited such support measures as benefitting their mental health. Another initiative is described in the excerpt below, namely the provision of “mindfulness sessions” (excerpt 8).

### Excerpt 8: UK interview 14

**Table:** 

01	INT:	Was there anything that you did to help you cope with any stress, like,
02		what kind of strategies that you might have used?
03	PAR:	*((3 lines of criticism of one of workplace’s measures removed))*
04	PAR:	And then we did mindfulness sessions and feedback, and the mindfulness
05		sessions I found were really good.
06	INT:	Why did you find them helpful?
**07**	**PAR**:	**I think for me**,** it was like my brain was like a hundred miles an hour**
**08**		**and just be able to calm and**,** like**,** get thinking about all the things**
**09**		***((inaudible))*what have happened**,** and just sort of like**,** let them go.**
**10**		**And it is what it is.**

The interviewer asks about coping strategies that the EOLC worker utilized to deal “with any stress.” After criticizing one of the measures that her workplace provided, the EOLC worker describes another measure, “mindfulness sessions.” She positively assesses this initiative. The mindfulness sessions “were really good” (line 05). The interviewer follows up (line 06) and the participant elaborates on the benefit of this initiative for her psychological state. The interviewee uses a simile to characterize her cognitive processes prior to undertaking the mindfulness sessions, “my brain was like a hundred miles an hour.” This simile reproduces culturally shared understandings of psychological distress—whereby, “calm” or “slow” minds represent a state of relative psychological wellbeing and stasis. Indeed, we can see such cultural understandings in the very names of popular mindfulness smartphone apps such as *Calm* [82]. She uses the idiomatic phrase “let them go” to describe ridding her ‘head’ of thoughts that we can infer are negative. Thus, it is through these mindfulness sessions that the EOLC worker constructs herself as achieving a positive psychological state.

The EOLC worker is describing an initiative that was provided by her organization. However, she also constructs this mental ‘calming down’ as achieved through her own actions. Indeed, she indexes “*my* brain” and thus implies her own agency in the process. Namely, that she managed to ‘calm’ her mind down through her own thinking and will by “letting” her negative thoughts “go.” Thus, these sorts of narratives show that organizational support mechanisms are constructed as also providing a means for EOLC workers to ‘help themselves’ in relation to their mental health, and to feel more positive about it.

## Discussion and conclusions

This paper focused on how EOLC workers in the UK and Hong Kong constructed positive psychological states. We found that EOLC workers foregrounded their positive psychological states and reformulated their negative states to positive ones. EOLC workers also dismissed the notion that their work had any negative psychological impact on them. Finally, we explored how interviewees constructed social and organizational support as mechanisms that improved their psychological states.

This work contributes to existing sociolinguistic research on mental health by forming part of an empirical shift in focus from its negative dimensions to its positive dimensions [11]. There has been much research focusing on how people describe mental *illness* and their *negative* mental health experiences. [6, 9] However, we made the argument earlier that mental health and psychological states are a spectrum of experiences. To restrict oneself to only focusing on the negative pole is limiting from an empirical perspective. This recent trend in discourse-based research parallels the emergence of the positive psychology movement [11, 12, 14]. Positive psychology emerged as an antidote to the negative orientation of psychology as a discipline, which is often concerned with studying “illness” [83] (p.6) and dysfunction. [84] Positive psychology has devoted itself to focusing on matters such as happiness, success, and flourishing [85, 86]. The point of our work is not to suggest a wholesale abandonment of discourse-based research on the negative aspects of mental health. Rather, the suggestion is for sociolinguistic and discourse-based research to widen their empirical scope and explore the construction of psychological wellbeing and resilience. In sum, research should focus on the *healthy* side of the mental health continuum.

Similarly, our work points to the importance of considering the multidimensional nature of mental health. The analytic focus of this paper was broad and encompassing insofar as mental health, feelings, emotions, and resilience were included under the rubric of “psychological states.” This analytic focus was grounded in existing epistemological and theoretical links. [13, 18, 20] Nevertheless, interviewees themselves also treated these dimensions as interconnected. For example, interviewees included terms and concepts like these in their answers to questions about their “mental health” and “mental wellbeing.” Thus, for participants, mental health is more than just a continuum with negative and positive poles that they might describe as “good” or “bad” mental health. Namely, for participants, mental health also appears to have affective dimensions, including feelings and emotions. As a first step, research should ground an understanding of mental health in people’s own meaning-making before progressing to a broader clarification of the theoretical and conceptual grounds for what we consider “mental health.” Ultimately, empirical research in this area should establish a shared consensus of what constitutes “mental health” in a discursive sense.

The findings show that people talk about psychological states and mental health in a range of different ways, or what one might call a “vocabulary of mental health”  [p.212] [87] or a “grammar of mental health talk” [p.266]. [88] EOLC workers explicitly described positive psychological states (i.e., “foregrounding the positive”). Nevertheless, a striking finding was that positive mental health was still made sense of, and constructed, in relation to its opposite—that is, negative mental health. For instance, one interviewee described herself as “not an anxious person” (Extract 5). This paper’s findings parallel other discourse-based research, such as Chang et al. (2022) who found that psychotherapists define mental health as including an “absence of negative emotions” [51] (p.1). Some of the “vocabulary of mental health” [87, p.212] identified in the analyses parallel existing research, such as disclosure [80, 81] and metaphors [74]. Nevertheless, it is not as simple as focusing on explicit descriptions of positive psychological states in discourse-based research on mental health. Research should expand its lexicon of mental health (so to speak) and focus on the more subtle lingusitic practices that people use to construct psychological states, including reframing, reformulation, and attributions of causality.

One limitation of this study is that the interviews were conducted during the COVID-19 pandemic. Thus, the narratives we examined may be unique to this crisis. However, some of the ways that EOLC workers constructed their psychological states may be generalizable beyond the pandemic. Indeed, some EOLC workers cited features of their work such as interpersonal relationships (Extract 2) as benefitting their mental health. Cultivating strong and mutual interpersonal relationships with patients and family members are essential aspects of EOLC. [89, 90] Thus, future research needs to examine how EOLC workers construct psychological states in a non-crisis context.

Another limitation of this work is that we did not directly compare cultural differences across the two samples. Relatedly, we did not examine the discursive strategies used in the six Cantonese interviews. These foci were beyond the scope of this paper. Our objective, in the first instance, was to consider how mental health was talked about similarly across two different cultural settings. The unique cultural forces at play in Hong Kong regarding mental health [91] may be manifest in how people talk about psychological states. Indeed, one paper in this special issue showed how Cantonese speakers would switch to English when talking about previous negative mental health experiences in wider narratives. [92] Examination of the cultural differences would likely elucidate unique discursive strategies that we did not identify here. In addition, such research could be beneficial in helping us broaden a discursive conceptualization of mental health beyond current Western-Anglocentric understandings. In sum, future research is necessary to tease out any cultural differences in the “grammar of mental health”. [88, p.266].

The COVID-19 pandemic upended the very nature of EOLC. Regardless of whether they were working in the UK or Hong Kong, EOLC workers spoke about the adverse mental health impacts associated with working during such tumultuous times. Yet, through such crises, these EOLC workers were still able to derive positive meaning from their work. Ultimately, this paper points to the immese resilience of these healthcare professionals and the importance of fostering positive psychological wellbeing in the workplace regardless of whether a pandemic is raging or not.

### Electronic supplementary material

Below is the link to the electronic supplementary material.

Supplementary Material 1

## Data Availability

The data used in this study and project are not available due to ethical restrictions.

## Footnotes

1 “Discourse-based research” involves studies that (a) utilize discourse analytic methods; (b) qualitatively examine talk and texts; and (c) identify socially constructed discourses of mental health.

2 The extracts from the Hong Kong interviews that we present in this paper are those conducted in English. Thus, the extracts are with non-native speakers. As such, there may be grammatical issues. We have not edited these out to be faithful to the original interviews.
