# Supplementary material for: “I’m not an anxious person”: end-of-life care workers constructing positive psychological states
Source: BMC Psychol. 2024 Aug 9;12:432. doi: 10.1186/s40359-024-01885-5 (PMC11316421; doi:10.1186/s40359-024-01885-5)
Supplement: Supplementary file 1 — Supplementary Material 1 [file 40359_2024_1885_MOESM1_ESM.docx]

**“I’m not an anxious person”: End-of-life care workers constructing positive psychological states**

*Semi-structured interview questions for Hong Kong end-of-life care workers:*

Background and demographics:

1. What is your job position?
2. How long have you worked in this position?
3. What is your educational background?

Working in end-of-life care:

1. Can you tell me about your work and duties?
2. What are the three best and worst things about your job?
3. Can you describe the ideal end-of-life care worker?

Death denial and stigma:

1. Can you tell me about your patients’ attitudes toward death?
2. Can you tell me about your own attitudes towards death and dying?

Communicating with patients and families:

1. What are the three easiest and hardest things about your conversations about death with patients and families?

Organizational matters:

1. Can you tell me about some of the difficulties you have at your workplace?
2. Can you tell me about the kinds of support your workplace provides for your wellbeing?

Well-being:

1. How has your job created problems for your well-being?
2. How has your wellbeing benefited from your job?
3. What are three strategies that you use to cope with job stress?

**“I’m not an anxious person”: End-of-life care workers constructing positive psychological states**

*Semi-structured interview questions for UK end-of-life care workers:*

- *Let interviewee sign and read consent form.*

Background and demographics:

1. What is your job position currently?
2. How long have you worked in your current position?
3. What is your educational background?
4. How long did you work in end-of-life care during the pandemic?

Working in end-of-life care in the pandemic:

1. Can you describe your job during the pandemic?
2. What were the three best and worst things about your job during the pandemic?
3. If you could go back and change anything about your work during the pandemic, what would you change?
4. How well do you think the United Kingdom dealt with the pandemic compared to other places around the world?

Communicating with patients and relatives in the pandemic:

1. What were the three easiest and hardest things about your conversations about death with patients and families during the pandemic?
2. How did the pandemic change how you communicated with patients and relatives?

The workplace in the pandemic:

1. Can you tell me about some of the difficulties you had at your workplace during the pandemic?
2. Can you tell me about the support your workplace provided for your wellbeing?

Well-being in the pandemic:

1. How did your work in the pandemic create problems for your well-being?
2. How did your work during the pandemic have positive effects on your wellbeing?
3. What were three strategies that you used to cope with stress during the pandemic?
